# Interactome of Arabidopsis Thaliana

**DOI:** 10.3390/plants11030350

**Published:** 2022-01-27

**Authors:** Merve Yilmaz, Merle Paulic, Thorsten Seidel

**Affiliations:** Dynamic Cell Imaging, Biochemistry and Physiology of Plants, Bielefeld University, 33501 Bielefeld, Germany; merve.yilmaz@uni-bielefeld.de (M.Y.); merle.paulic@uni-bielefeld.de (M.P.)

**Keywords:** protein–protein interaction, *Arabidopsis thaliana*, interactome, Cytoscape

## Abstract

More than 95,000 protein–protein interactions of *Arabidopsis thaliana* have been published and deposited in databases. This dataset was supplemented by approximately 900 additional interactions, which were identified in the literature from the years 2002–2021. These protein–protein interactions were used as the basis for a Cytoscape network and were supplemented with data on subcellular localization, gene ontologies, biochemical properties and co-expression. The resulting network has been exemplarily applied in unraveling the PPI-network of the plant vacuolar proton-translocating ATPase (V-ATPase), which was selected due to its central importance for the plant cell. In particular, it is involved in cellular pH homeostasis, providing proton motive force necessary for transport processes, trafficking of proteins and, thereby, cell wall synthesis. The data points to regulation taking place on multiple levels: (a) a phosphorylation-dependent regulation by 14-3-3 proteins and by kinases such as WNK8 and NDPK1a, (b) an energy-dependent regulation via HXK1 and the glucose receptor RGS1 and (c) a Ca^2+^-dependent regulation by SOS2 and IDQ6. The known importance of V-ATPase for cell wall synthesis is supported by its interactions with several proteins involved in cell wall synthesis. The resulting network was further analyzed for (experimental) biases and was found to be enriched in nuclear, cytosolic and plasma membrane proteins but depleted in extracellular and mitochondrial proteins, in comparison to the entity of protein-coding genes. Among the processes and functions, proteins involved in transcription were highly abundant in the network. Subnetworks were extracted for organelles, processes and protein families. The degree of representation of organelles and processes reveals limitations and advantages in the current knowledge of protein–protein interactions, which have been mainly caused by a high number of database entries being contributed by only a few publications with highly specific motivations and methodologies that favor, for instance, interactions in the cytosol and the nucleus.

## 1. Introduction

Most proteins do not act alone but interact with others to fulfill a number of processes and functions within a cell [1]. For this reason, they often form protein complexes and molecular machines to facilitate and enable their proper function for the cell [1,2,3]. Protein–protein interactions (PPI) are understood as physical contact, and molecular attaching between proteins and are a prerequisite for protein functioning in living cells [4]. Therefore, a better understanding of proteins and their respective function is facilitated by identifying protein–protein interactions. Analyzing and characterizing PPI contributes to an understanding of proteins’ activity and response to biochemical, signaling and functional processes [1].

PPIs are essential or responsible for a broad spectrum of processes and functions, such as cell–cell interactions, the transformation and transportation of information through the cell and different transportation processes of molecules within a cell, such as the transport of ions, electrons and protons [5]. For instance, an impressive interaction of multiple proteins cooperates in mitochondria: electrons are transported to generate a proton motif force at the respiratory chain, which is used for ATP generation and includes the interaction of four major complexes [6,7]. Looking at the regulation of protein functions, the binding of calcium-binding EF-hand proteins, 14-3-3 proteins and thioredoxins acts as a cellular regulator [8,9,10].

Although there are a variety of methods to identify PPI, classical techniques comprise yeast-2-hybrid and mass spectrometry (MS) [11,12,13]; indeed, it is the development of these two methods that contributed the most to progress in identifying protein–protein interactions [11]. Whereas the classical yeast-2-hybrid assay depends on nuclear import of the interaction partners to enable transcriptional activation of the reporter, the mating-based split-Ubiquitin system (mbSUS) allows testing of interactions between membrane proteins, since a transcription factor is released by Ubiquitin-dependent proteases following bimolecular complementation of Ubiquitin [14,15]. However, one has to keep in mind that both approaches rely on a heterologous expression system, with its typical advantages and disadvantages regarding, for instance, the localization and developmental regulation of protein expression. Isolating complexes out of plant tissues or cells by co-immunoprecipitation or tandem affinity purification (TAP) ensures that the complexes have been exposed to their native environment while isolated [16,17], with the advantage of fewer false positives due to the tandem-affinity purification, despite the requirement for tagged bait proteins in the case of TAP [17]. In vivo assays such as Förster resonance energy transfer (FRET) between fluorescent proteins, or the bimolecular fluorescence complementation of fluorescent proteins, allow for the detection of protein–protein interactions in a homologous expression system, though many of these experiments have been performed subsequent to transient expression in protoplasts or epidermal cells of tobacco leaves [18,19].

Experimental data contribute to online data resources through collection, analysis and visualization, with computational methods even enabling predictions based on the deposited data. In fact, these methods have grown significantly in popularity over the past few years [3,11,20,21]. The entity of PPI within a living organism or particular cell is called the “interactome” [11,22,23,24]. Studying the interactome is crucial to understanding how cellular interactions are organized [11]. There is an increasing interest in this field, moving from the elucidation of genomes to working on proteomes and interactomes and respective databases [25,26,27,28]. With the help of computational methods, databases of interactomes for several species were launched (e.g., *Escherichia coli* [29,30,31], *Saccharomyces cerevisiae* [12,32,33,34], *Drosophila melanogaster* [35] as well as *Caenorhabditis elegans* [36]). In recent years, several approaches have dealt with the interactome of the plant *Arabidopsis thaliana.* This article aims at tracing and visualizing protein–protein interactions of *A. thaliana* and combines data from multiple databases within a network to reveal limitations and advantages in the current knowledge of the Arabidopsis interactome.

## 2. Results

According to TAIR, the genome of *Arabidopsis thaliana* contains 27,416 protein-coding genes (Table S3). The main portion of PPIs was identified in several databases, while an additional 920 PPIs were identified through a PubMed search for studies of interactions in Arabidopsis published over the last twenty years [37,38,39,40,41,42,43,44,45,46,47,48,49,50,51,52,53,54,55,56,57,58,59,60,61,62,63,64,65,66,67,68,69,70,71,72,73,74,75,76,77,78,79,80,81,82,83,84,85,86,87,88,89,90,91,92,93,94,95,96,97,98,99,100,101,102,103,104,105,106,107,108,109,110,111,112,113,114,115,116,117,118,119,120,121,122,123,124,125,126,127,128,129,130,131,132,133,134,135,136,137,138,139,140,141,142,143,144,145,146,147,148,149,150,151,152,153,154,155,156,157,158,159,160,161].

In total, 95,382 protein–protein interactions (PPI) were identified for 12,617 proteins, corresponding to approximately 46% of the number of genes coding for proteins (Tables S1 and S2). However, it should be considered that this number of genes does not account for splice variants. Co-expression data were available for 45,427 protein pairs, corresponding to 48% of the PPI in the network. In the IntAct-database we also found PPI-data for ten other plant species (including green algae *Chlamydomonas reinhardtii*) with comparatively low numbers of interactions (Table 1). Data from the STRING database were omitted due to the high fraction of predicted interactions [162].

**Figure 1:**
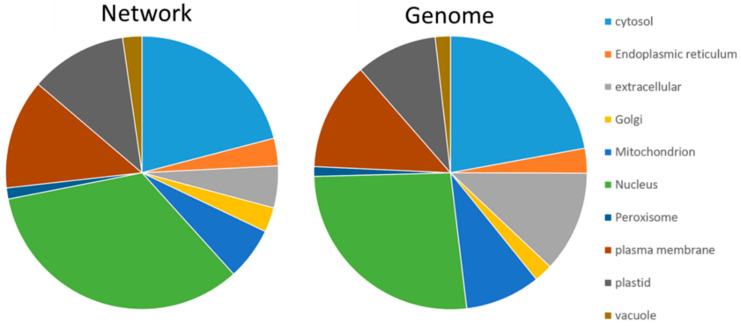
Subcellular distribution of proteins in the network and according to protein-coding AGI listed in the genome. The diagram is based on the consensus prediction provided by SUBA4. Nuclear proteins (green) were enriched in the network, and a depletion was visible for extracellular (grey) and mitochondrial proteins (blue).

The consensus subcellular localization of SUBA was applied to have a first look at the contribution of compartments (Tables S4–S9). This revealed an enrichment of nuclear proteins (26% to 34%) and a depletion of extracellular (12% to 5%) and mitochondrial proteins (9% to 6%) (Figure 1).

**Figure 2:**
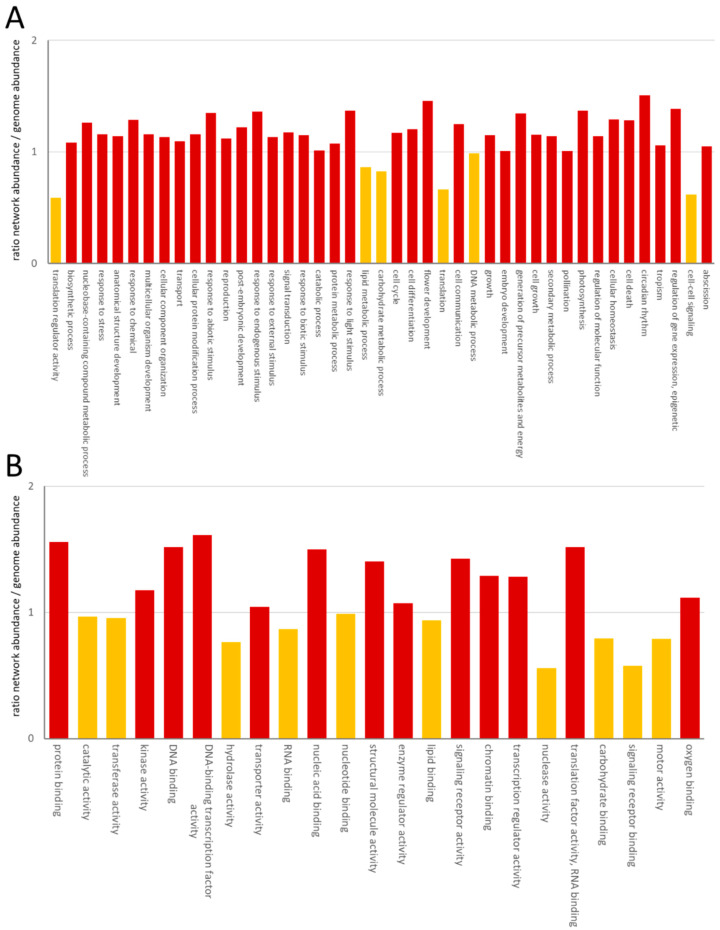
Enrichment of GO category process (**A**) and GO category function (**B**). The ratio has been calculated as the number of GOs in the network/the number of GOs of protein-coding genes in *A. thaliana*. GOs with a ratio >1 were considered as enriched in the network and labeled in red. Yellow columns correspond to ratios <1.

The GO distribution confirmed the high content of nuclear proteins through enrichment of proteins involved in DNA-binding, transcription factor activity, nucleic acid binding, chromatin binding and transcription regulator activity (Figure 2).

Among the process GOs, a depletion of proteins involved in translation was obvious. Although one large-scale experiment focused on the interaction network of membrane proteins, we could only observe limited enrichment in plasma-membrane, ER, vacuolar or Golgi-proteins, which increased from 18% to 21%. The process and function categories showed a consistently reduced abundance in the lipid metabolism process and lipid-binding function, as well as a reduced cell–cell signaling process and signaling receptor binding function (Figure 2). Reduction in the processes of translation regulator activity and translation was accompanied by a reduction in the RNA-binding function (Figure 2).

The network was constructed based on UniProt-IDs. For enhanced identification, commonly used AGIs were used as node labels, with the node color providing information on subcellular localization, which corresponds to the consensus localization provided by SUBA4 (Network S10). The network also contains experimental data on the subcellular localization, which were available for 9207 and 3162 proteins as mass spectroscopy and GFP data, respectively. Biochemical data were included as node attributes; for instance, TAIR provided the number of transmembrane helices for 725 proteins.

Subnetworks were defined by subcellular localization and GO-terms/gene descriptions and contain neighboring proteins/nodes of the selected nodes. Using this definition, ten subnetworks contain compartment-specific data. The respective number of interactions varied between the organelles. The largest subnetworks were associated with cytosolic and nuclear proteins (Table 2).

The organelle-subnetworks contain the following respective keyplayers: the ER network contains components of the COPII-vesicle coat (Sec12, Sec23/24, Sec13), protein sorting (cornichons and p24-proteins; Rer1) and proteins for protein biosynthesis, such as the Get-complex and ERAD-related proteins (IRE1, DERLIN1). The Golgi network comprises typical Xylosyl-, oligosaccharyl-, galacturonosyl-transferases, glucuronyltransferases, hydroxyproline O-galactosyltransferase, N-acetylglucoseaminyl transferase I, Golgi-SNAREs Gos1 and Gos11. The vacuolar network contains seven V-ATPase subunits, a V-PPase and several other vacuolar transporters. Keyplayers of respiration were found in the mitochondria, including subunits of complexes I–IV and alternative oxidases, but also subunits of the TOM-complex, mitochondrial RNA-editing enzymes and mitochondrial ribosomal proteins. Catalases, catalase chaperones, photorespiratory enzymes and peroxin 11 are present in the peroxisomal network. The chloroplast network contains key players of photosynthesis, such as D1 and D2, CP43 and CP47; subunit IV of the cytochrome b6/f complex; PsaF, PsaG, PsaL, PsbP-proteins; PsbQ; ferredoxin-NADP(H) oxidoreductases and proteins of the light-harvesting complexes; but also cytosolic, nuclear and plasma membrane proteins. It is remarkable that a high content of plasma membrane and nuclear and cytosolic proteins was found in all organelle subnetworks. The nuclear network is characterized by DNA- and RNA-modifying enzymes, such as polymerases, helicases, polyadenylation, ribonucleases, histones and enzymes of histone modification; such as E3 ubiquitin ligases, histone chaperones, histone methylation complex and histone acetyltransferases; while enzymes for deacetlyation, transcription factors and other transcription-regulating factors were also found in this subnetwork. Last but not least, a large variety of proteins were present in the cytosolic subnetwork, reflecting its involvement in many processes: components of the translational machinery (ribosomal proteins, elongation factors, chaperones); of signal transduction (calmodulins, thioredoxins, 14-3-3 proteins, kinases, phosphatases, enzymes of ubiquitinylation and sumoylation) and subunits of the proteasome contribute to the cytosolic network of protein–protein interactions.

The process-related subnetworks show a high contribution of protein biosynthesis (transcription and translation) to the number of protein–protein interactions, although the number of proteins with a function in translation was reduced in the network (Table 3). Conserved and fundamental processes, such as vesicle transport, glycolysis and respiration, were characterized by a surprisingly low level of representation in the network (Table 3).

Some proteins showed an apparent sticky behavior with vastly more interaction counts than other protein family members. This includes the ER cargo receptor Cnih1 (AT3G12180), which interacts with 648 out of 662 Cnih-interaction partners, in comparison to its isoforms Cnih3 (AT1G62880) and Cnih4 (AT1G12390), which count eight and ten interacting proteins, respectively. Ubiquitin 3 (UBQ3) showed a similar behavior by interacting with 1317 proteins out of 2937 in the Ubiquitin subnetwork.

**Figure 3:**
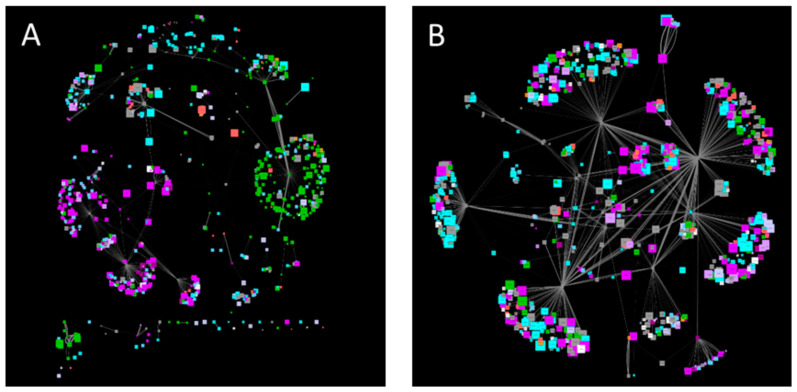
Subnetworks of Thioredoxins (**A**) and 14-3-3 proteins (**B**). Node color corresponds to subcellular localization (Cytosol—grey, nucleus—cyan, plastid—green, mitochondrion—red, peroxisomes—orange, ER—light blue, Golgi—light purple, vacuole—dark purple, plasma membrane—purple, extracellular—white), node size indicates the molecular weight. Edge size displays co-expression strength.

Looking at signaling subnetworks revealed organelle-specificity for thioredoxins with plasma membranes and plastidic clusters, while 14-3-3 proteins showed no organelle specificity (Figure 3). Furthermore, the 14-3-3 subnetwork contains primary active transporters such as proton pumps and calcium pumps, pointing to a role of 14-3-3 proteins in regulating calcium and pH-homeostasis, as recently suggested for pH [163].

The SNARE network contains members of the tethering complex exocyst and cell plate assembly TRAPPII tethering factors, heavy chain subunits of clathrin-coated vesicles and SNAP25-like proteins, α-SNAP proteins and members of the GET-complex, all of which are tightly connected to the function of SNAREs—despite an overrepresentation of plasma membrane proteins. The Ubiquitin subnetwork reflects the multiple functions and sticky behavior of Ubiquitin, with more than 4500 interactions in the network.

**Figure 4:**
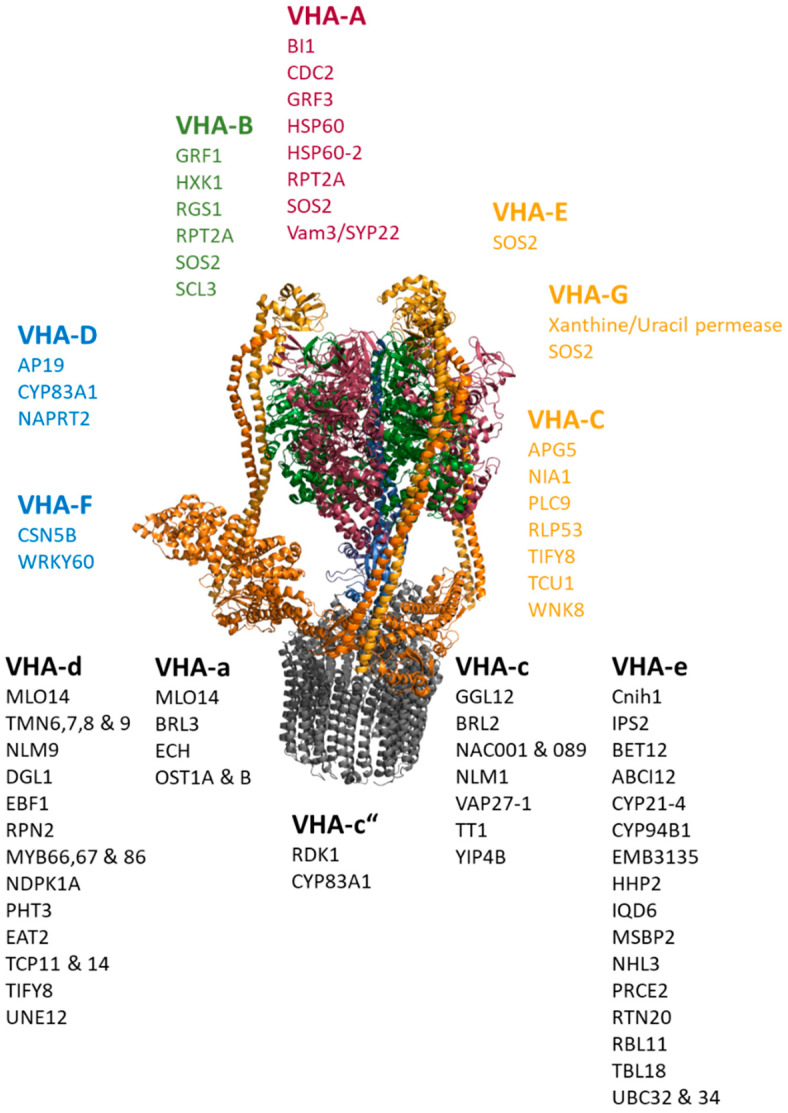
Protein–protein interactions of V-ATPase subunits. The catalytic head comprises VHA-A (red) and VHA-B (green). Subunits of the peripheral stalk (VHA-C, VHA-E, VHA-G, VHA-H) are given in orange-yellow, and the central stalk (VHA-D, VHA-F) is shown in blue. The membrane integral sector V_0_ consists of VHA-a, the proteolipids VHA-c and VHA-c”, the bearing VHA-d and the subunit VHA-e. A selection of interacting proteins is given per subunits, regardless of the isoform.

Next, the network was applied to gain new insights into the interactions of the plant proton-translocating ATPase of the vacuolar type (V-ATPase) and thereby to test the usefulness of the network for addressing biological questions. The V-ATPase is a multi-subunit proton pump, which transduces conformational alterations due to ATP-hydrolysis within the catalytic subunits VHA-A into rotation of the central stalk formed by VHA-D and –F. VHA-d transfers the rotation to the proteolipids VHA-c and VHA-c”. These bear the proton binding sites, while half channels of VHA-a C-terminus serve as cytosolic proton inlet and luminal exhaust. VHA-a’s N-terminal domain contributes to a stator structure together with VHA-C, -E, -G and –H, stabilizing and fixing the catalytic head to the membrane [163]. For instance, the analysis aimed to provide evidence for the existence of a Golgi-dependent transport route to the vacuole and involvement of 14-3-3 proteins in V-ATPase regulation, which might indicate a light-dependent regulation, as reported for barley [164]. The V-ATPase subnetwork contained 448 interactions. Nearly all subunits were listed as interacting with the plasma membrane intrinsic proteins PIP1B, PIP2A, Ubiquitin UBQ3 and the ammonium transporter AMT1;3, probably originating from experiments that did not distinguish between the subunits. The provided subcellular localization was applied to omit proteins of the chloroplast and mitochondria from subsequent analyses. According to the previously mentioned ER-export and transport routes along the secretory pathway, the data revealed an interaction between VHA-e and the ER exit site cargo receptor Cornichon 1 (Cnhi1) with a high co-expression level and between VHA-e1 and the ER to Golgi SNARE BET12. Interactions with SNAREs were also observed for VHA-A and the vacuolar SYP22, while the interaction of VHA-c and VAP27-1 links the V-ATPase subsector V_0_ to ER-plasma membrane tethering, and VHA-D interacts with an adapter protein (AP19) of Clathrin-coated vesicles. On the regulatory level, interactions with 14-3-3 proteins were found for VHA-A and GRF3 as well as for VHA-B and GRF1 (Figure 4).

Interaction between the hexokinase 1 and the glucose receptor RGS1 with VHA-B points to an energy-dependent regulation that acts directly on the head structure of the V-ATPase, which catalyzes the ATP-hydrolysis. Furthermore, VHA-A and VHA-B (as well as VHA-E and G) are targets of salt overly sensitive 2 (SOS2), a Ca^2+^-sensor with a role in K^+^-homeostasis and salinity. V-ATPases also interact with the Calmodulin-binding IDQ6, but with a low co-expression level and steroid-binding proteins such as MSBP2 and RBL11. The nucleoside diphosphate kinase NDPK1A, which modulates the auxin-transport, interacts with VHA-c, while the soluble kinase Without No Lysine 8 (WNK8) interacts with VHA-C. Interestingly, VHA-C binds to APG5, which is responsive to nitrogen starvation, and to the nitrate reductase NIA1. A couple of transcription factors were reported to interact with V-ATPase subunits, including three members of the MYB-family, two of the TCP-family, two of the NAC-family, UNE12, scarecrow-like 3, transparent testa 1 and WRKY60.

The interaction of V-ATPase subunits and proteins involved in cell wall synthesis, such as EMB3135, TBL18, ECH and YIP4B, point to a function of V-ATPase in cell wall synthesis. Here, V-ATPase joins the complex of ECH and YIP4B at the TGN via VHA-a and VHA-c.

The interaction between V-ATPase and other transporters is also apparent, where the interaction of the phosphate transporter PHT3 and the phosphate-starvation-induced IPS2 with V_0_-subunits might correspond to the terminal signal-transduction (Figure 4).

## 3. Discussion

**Figure 5:**
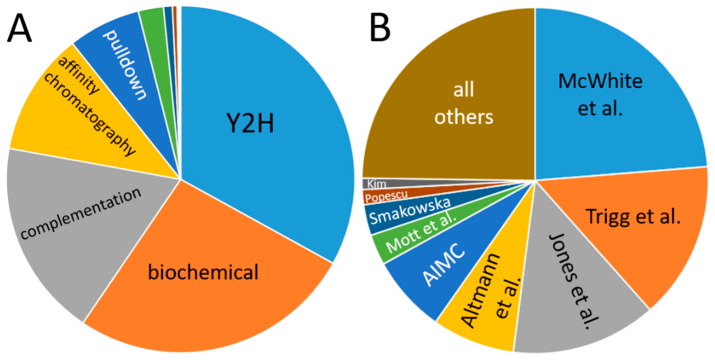
Contribution of individual methods (**A**) and publications (**B**) to the databases. (**A**) “Biochemical” denominates data from co-fractionation/mass spectroscopy (CF/MS) [167]. Enzymatic studies (green), Förster resonance energy transfer (dark blue), genetic interference (brown), X-ray experiments, imaging techniques and FarWestern contribute a comparatively low amount of data. (**B**) Publications are given by the name of the first author; Smakowska corresponds to Smakowska-Luzan [165,166,168,169,170,171,172,173,174].

Data in the databases are dominated by yeast-2-hybrid experiments, and the main portion has been contributed by only a few publications (Figure 5): the share of yeast-2-hybrid experiments is approximately one-third, and four publications account for more than half of the data. This creates a bias in the data, which results from the aim of the studies and their methodical limitations. For instance, one of the dominant studies involved screening for transcription factor interactions [165], while another focused on interactions of membrane proteins [166] so that the observed enrichment of nuclear proteins and plasma membrane protein interactions is plausible.

The experimental design has an additional impact on the subcellular localization of the proteins in the network: yeast-2-hybrid relies on protein–protein interactions in the nucleus or applies artificial nuclear localization sequences. The mating-based split-Ubiquitin system works for proteins with cytosolic C-termini in particular for the C-terminal half of Ubiquitin due to the fusion with a transcription factor, which is released by Ubiquitin-dependent proteases. The N-terminal half of Ubiquitin (Nub) can be fused to either a cytosolic N- or C-terminus [175]. For instance, mitochondrial proteins cannot be addressed by these methods, potentially contributing to a low representation of mitochondrial proteins in the network.

Another consideration applies to the probability of an interaction; complementation assays such as bimolecular fluorescence complementation and mbSUS are often performed in heterologous expression systems, partially with strong promoters. This uncouples protein–protein interactions from their spatial and temporal specificity in the plant. The specificity is widely ensured by pulldown experiments, co-immunoprecipitations, affinity chromatography and genetic interference, since proteins are mostly under control under their endogenous promoters and in their native environment. However, to compensate for the problem of low specificity and the risk of false-positive interactions in the network, the data were supplemented by the co-expression lsMR-values as a measure of probability. The higher the co-expression, the higher the probability of a protein–protein interaction in the plants. Low co-expression might then be an indicator of false-positive interactions. However, many proteins with related functions were found in the expected and typical clusters, demonstrating the value of the databases for proposing new hypotheses. The visible crosstalk between signaling pathways, such as 14-3-3-protein-mediated signaling and calcium signaling, or the control of proton pumps by 14-3-3 proteins, reveals the complexity of regulation and signal transduction in the plant.

Interaction between V-ATPase subunit VHA-A and 14-3-3 proteins has been shown to be blue-light-dependent in barley [164], and the observed interaction of 14-3-3 proteins and VHA-A/-B points to similar regulation in *A. thaliana*, which links the V-ATPase activity to daylight. In this context, the observed interplay of V-ATPase and nitrogen assimilation and availability fits well into light-dependent activation. A glucose-dependent regulation by reversible dissociation of the V-ATPase complex has been described for the yeast and the mammalian V-ATPase [176], but could not be observed in plants [177]. Instead, VHA-B1 was reported to interact with hexokinase 1 in the nucleus as part of the glucose-dependent gene-regulation complex [178]. However, this interaction was observed for VHA-B1 in the absence of other VHA-subunits, describing a moon-lighting function of VHA-B1. Interestingly, interaction with glycolytic aldolase was not found in the dataset, though its regulatory function has been described for the V-ATPase in rice [179]. The interaction with the glucose receptor RGS1 might be an alternate way to sense the glucose level in order to regulate the V-ATPase and its ATP consumption.

On the cell-biological level, the data show a clear linkage to cell wall synthesis and is in good agreement with the previously reported cell wall defects of V-ATPase knock out lines affected in the TGN [180]. Actually, the PPI-data indicate a contribution of V-ATPase to the TGN-located complex of ECHIDNA and the YPT/Rab-interacting protein 4B, which is essential for the secretion of cell wall saccharides.

Last but not least, the mechanism of the transport of V-ATPases is less understood. V_0_-subunits such as VHA-a and VHA-c were observed to be transported on a fast track from the ER to the vacuole and thus bypassed the Golgi and TGN, while the V_1_-subunit VHA-E followed the canonical secretory pathway [181,182,183]. The interaction with components of the ER to Golgi transport, such as Cnih1 and Bet12, supports the finding of COP II-dependent transport without ruling out an alternate direct pathway to the vacuole. Furthermore, the interaction found between V-ATPase subunit VHA-c and VAP-27-1 even links the V-ATPase to contact sites between the ER and the plasma membrane [184].

## 4. Materials and Methods

Databases—all gene descriptions and GO-terms were bulk downloads from The Arabidopsis Information Resource (TAIR, www.arabidopsis.org, accessed on 16 July 2021). First, a list of all Arabidopsis genome identifiers (AGI) was downloaded from TAIR and used for interaction searches in IntAct and Biogrid. If required, UniProt-IDs were mapped to AGI using the UniProt ID-mapping (https://www.uniprot.org/uploadlists/, accessed on 16 July 2021).

PubMed (https://pubmed.ncbi.nlm.nih.gov/, accessed on 16 July 2021) and Web of Science (https://www.webofscience.com/wos/woscc/basic-search, accessed on 16 July 2021) were applied to screen the literature for protein–protein interactions. Keywords were *Arabidopsis thaliana*, protein interaction and one of the following methods: yeast-2-hybrid, mating-based split-Ubiquitin system, co-immunoprecipitation, TAP-tag, BiFC. The time span of publication dates was 2002–2021.

Data on subcellular localization were obtained from SUBA4 (https://suba.live, accessed on 16 July 2021). Co-expression data came from ATTED II (https://atted.jp, accessed on 16 July 2021). Data were obtained 21 July 2021.

Networks—required data sheets were created using Microsoft Excel (Tables S1–S9); all networks were designed using Cytoscape version 3.8.2 (Network S10). Simple grid layout was chosen for the entire master network, compound spring embedder layout for sub-networks. LsMR-values were chosen as edge-attributes; all other data were node attributes. Cytoscape styles were defined to express the co-expression, subcellular localization and molecular weight (Table 4).

## 5. Conclusions

The dataset contains known protein–protein interactions in *A. thaliana*, which were combined with expression, subcellular localization and GO-association data. Interactions can be screened by gene identifiers and found pairs evaluated based on co-expression and localization data to estimate the probability of interaction. The exemplary analysis of the V-ATPase revealed interactions that were well described in the literature but also others that have not been considered before. Such novel interactions might be worthwhile to investigate further in order to open new perspectives on the characterization of a protein or complex.

## Figures and Tables

**Table 1:** Protein–protein interactions of other plant species as deposited in IntAct.

Plant Species	Number of Interactions
*Arabidopsis thaliana*	81,399
*Zea mays*	18
*Ricinus communis*	5
*Chlamydomonas reinhardtii*	17
*Glycine max*	46
*Solanum tuberosum*	3
*Oryza sativa*	348
*Nicotiana tomentosiformis*	3
*Solanum lycopersicum*	141
*Sorghum bicolor*	1
*Vitis vinifera*	1

**Table 2:** Compartment-specific subnetworks.

Network	Number of Interactions	Percentage
Master network	95,382	100%
Cytosol	7678	8%
Nucleus	10,001	10%
Plastid	4387	5%
Mitochondrion	3672	4%
Peroxisome	914	1%
ER	2800	3%
Golgi	2598	3%
Vacuole	1957	2%
Plasma membrane	6681	7%
Extracellular	2838	3%

**Table 3:** Process-related subnetworks.

Network	Number of Interactions	Percentage
Master network	95,382	100%
Photosynthesis	206	<1%
Transcription	24,855	26%
Translation	10,035	11%
Respiration	273	<1%
Glycolysis	197	<1%
14-3-3 network	1419	1%
Calmodulin	1056	1%
Thioredoxin	1175	1%
Ubiquitin	4705	5%
SNARE	1830	2%
Rab-GTPase	619	<1%
Clathrin	332	<1%
COP I	232	<1%
Cornichon	1089	1%

**Table 4:** List of applied Cytoscape styles.

Target of Style	Attribute Group	Attribute	Style
Node	Subcellular localization (discrete mapping)	Cytosol	Grey
		Nucleus	Cyan
		Plastid	green
		Mitochondrion	Red
		Peroxisomes	Orange
		ER	Light blue
		Golgi	Light purple
		Vacuole	Dark purple
		Plasma membrane	Purple
		extracellular	White
Node	Protein properties (continuous mapping)	Molecular weight	Node size
Edge	Co-expression (continuous mapping)	LsMR	Line size

All collected data are given in Excel sheets as Supplementary Materials.

## Data Availability

No new data were created or analyzed in this study. The data presented in this study are available in supplementary material here.

## Supplementary Materials

The following are available online at https://www.mdpi.com/article/10.3390/plants11030350/s1, Table S1: node attributes.xlsx, Table S2: edge attributes.xlsx, Table S3: list of AGIs.xlsx, Tables S4–S9: SUBA4-data, Network S10: Cytoscape network file Arabidopsis PPI network.cys.
